# The Correlation between the CLEC16A Gene and Genetic Susceptibility to Type 1 Diabetes in Chinese Children

**DOI:** 10.1155/2012/245384

**Published:** 2012-06-20

**Authors:** Yanmei Sang, Wei Zong, Jie Yan, Min Liu

**Affiliations:** National Key Discipline of Pediatrics, Ministry of Education and Department of Endocrinology, Beijing Children's Hospital, Capital Medical University, Beijing 100045, China

## Abstract

*Objective*. The CLEC16A gene is related to the genetic susceptibility to T1DM with racial variability. This study investigated the association between CLEC16A gene polymorphisms and T1DM in Chinese children. *Methods*. 131 Chinese children with T1DM were selected for study, and 121 healthy adult blood donors were selected as normal controls. PCR and mass spectrometry was used to study the distributions of 17 CLEC16A alleles in patients and controls. The relationship between CLEC16A gene polymorphisms and T1DM was studied. *Results*. The distributions of two polymorphisms (rs12921922, rs12931878) of CLEC16A in T1DM and healthy controls were significantly different, while the distributions of other CLEC16A polymorphisms show no significant differences. The alleles of rs12921922 are C and T. The frequency of the T allele was significantly increased in patients versus healthy controls. The alleles of rs12931878 are A and C. The frequencies of the A allele are significantly increased in T1DM patients versus healthy controls. *Conclusion*. Two polymorphisms in the CLEC16A gene correlate with increased susceptibility to T1DM in Chinese children, revealing that it was another new gene that correlates with susceptibility to T1DM in multiple populations.

## 1. Introduction

Type I diabetes (T1DM) is a polygenic chronic autoimmune disease, and to date, more than 50 genes has been found to be related to the disease [1]. Among these genes, the correlation between susceptibility to T1DM and the HLA, PTPN22, CTLA4, and insulin genes have been proven in different ethnic groups [2–4]. In 2007, Hakonarson et al. [5] conducted a genomewide association study to identify novel T1DM susceptibility genes in a large sample of European descendants. Their results further confirmed the previously identified T1DM susceptibility genes. At the same time, the results also demonstrated the correlation between T1DM and the variation of a 223Kb fragment in chromosome 16p13. This fragment only contains the CLEC16A gene (also known as KIAA0350), indicating that the noted genetic variation comes from this gene. The full length of the CLEC16A gene is 237,597bp, containing 24 exons. The results from Hakonarson et al. [5] showed that three loci in the noncoding area of CLEC16A gene, namely rs2903692, rs725613, and rs17673553, were significantly correlated with genetic susceptibility to T1DM. In 2009, Awata et al. [6] revealed that T1DM pathogenesis is also related to the CLEC16A gene in the Japanese population. These results suggested a relationship between the CLEC16A gene and the pathogenesis of T1DM. In this study, the correlation between CLEC16A gene and the genetic susceptibility of type 1 diabetes in Chinese children was investigated in 131 Chinese children with T1DM using time-of-flight mass spectrometry techniques. (Technical support was provided by Bioyong Technologies Inc., Beijing.) We aimed to elucidate the relationship between the CLEC16A gene and the genetic pathogenesis of T1DM in Chinese children.

## 2. Data and Methods

### 2.1. General Information

From August 2006 to February 2009, 131 unrelated children with T1DM who were treated at Beijing Children's Hospital were recruited. The median age was 8.6 years (*P *_25_–*P *_75_: 2.4–11 years). There were 69 boys and 62 girls. All patients met the 1999 WHO diagnostic criteria for type 1 diabetes [7]. Meanwhile, 121 healthy unrelated adult blood donors were recruited as controls and all signed consent forms. The controls had no history or family history of diabetes or autoimmune diseases.

### 2.2. Methods

#### 2.2.1. Primers

The primers and reaction conditions for multiplex polymerase chain reaction (PCR), as shown in Table 1 and single-base extension reaction were designed using iPLEX GOLD technology and Sequenom MassARRAY molecular array platform.

#### 2.2.2. Polymerase Chain Reaction

Multiplex PCR was performed with the PCR primers designed by iPLEX GOLD, and the reaction adopted the conditions suggested by iPLEX GOLD. Specific reaction conditions were as follows: the total reaction volume for the PCR reaction was 5 *μ*L, including 1.75 *μ*L of sterilized distilled water, 0.625 *μ*L of 10X PCR buffer (containing 15 mM MgCl_2_), 0.325 *μ*L of 25 mM MgCl_2_, 0.1 *μ*L of 25 mM dNTP Mix, 1 *μ*L of 0.5 *μ*M Primer Mix, 0.2 *μ*L of 5 U/*μ*L HotStar Taq, and 1 *μ*L of DNA. After denaturing at 94°C for 15 minutes, the reaction underwent the following thermal cycles: denaturation at 94°C for 20 minutes, annealing at 56°C for 30 seconds, and extension at 72°C for 1 minute. After 45 cycles, the products were further extended at 72°C for 3 minutes.

#### 2.2.3. Human Shrimp Alkaline Phosphatase (SAP) Enzyme Digestion

Residual dNTP in the PCR reaction was digested by SAP enzyme to prevent interference with the subsequent single-base extension reaction. Specific procedures and reaction conditions were as follows: the total volume of enzyme digestion reaction was 2 *μ*L, including 1.53 *μ*L of sterilized distilled water, 0.17 *μ*L of 10X SAP buffer, and 0.3 *μ*L of SAP enzyme. The reaction mixture was placed in 37°C water bath for 40 minutes, followed by 85°C water bath for 5 minutes.

#### 2.2.4. Single-Base Extension Reaction

With the single-base extension PCR primers designed by iPLEX GOLD, the dNTP in routine PCR reaction was reduced to ddNTP. Single-base amplification was then performed under the PCR conditions suggested by iPLEX GOLD. Specific procedures and conditions were as follows. The total reaction volume was 2 *μ*L, including 0.755 *μ*L of sterilized distilled water, 0.2 *μ*L of 10X iPlex buffer, 0.2 *μ*L of iPlex termination buffer, and 0.041 *μ*L of iPlex enzyme. After denaturing at 94°C for 30 seconds, the reaction underwent the following thermal cycles: denaturation at 94°C for 5 seconds, annealing at 52°C for 5 seconds, and extension at 80°C for 5 seconds. In each cycle, the annealing and extension steps were repeated 5 times. After 40 cycles, the products were further extended at 72°C for 180 seconds, and the products were then purified. After the mass system was calibrated by standards provided by Sequenom, the samples were loaded into time-of-flight mass spectrometry, and data was collected automatically for further analysis.

### 2.3. Statistical Analysis

Quantitative data were examined by an *χ* ^2^ test, and the significance level *α* was set at 0.05. The 95% Confidence Intervals (CIs) of allele frequency were calculated. A logistic regression formula and software in SPSS11.5 for Windows were used for the analysis.

## 3. Results

Among the 17 loci with a single-nucleotide polymorphism in the CLEC16A gene, two loci (rs12921922 and rs12931878) showed a significant difference in frequency distribution between the T1DM and healthy control groups. The frequency distribution of the remaining 15 loci showed no significant difference between T1DM and healthy control groups.

## 4. Discussion

Type 1 diabetes (T1DM) is a polygenic chronic autoimmune disease which results from the interaction of genetic and environmental factors. In the past, studies have shown that the HLA-complex, CLTA4, insulin, and PTPN22 genes are involved in the genetic pathogenesis of T1DM [8]. Being worthy to be mentioned is, large sample studies revealed that, of the Europeans loci related to T1DM, PTPN22 is the only one that has not been replicated in East Asians. Recently, a genome-wide association study (GWAS) of Caucasians conducted by WTCCC (Wellcome Trust Case Control Consortium) made new breakthroughs in the understanding of the genetic pathogenesis of T1DM. Using genome-wide association study technology, single-nucleotide polymorphisms in the range of 500kb (Affymetrix gene chip) and single-nucleotide polymorphisms in the range of 550kb were studied [9], and a novel chromosomal region 16p13, which may be related to T1DM, was identified.

CLEC16A is the only gene in the 233kb fragment of chromosome 16p13. The gene product of CLEC16A is one of the C-type lectins, but the function of the encoded protein is unknown. Studies have shown that this gene product is expressed in almost all immune cells, including dendritic cells, B lymphocytes, and natural killer cells, cells which play an important role in the pathogenesis of T1DM. The broad expression of the CLEC16A gene product in those immune cells suggests that a genetic mutation of CLEC16A could participate in the pathogenesis of T1DM through modification of immunological functions [10, 11].

Awata et al. [6] found that T1DM pathogenesis is also related to the CLEC16A gene in the Japanese population. In their genome-wide association study (GWAS), the CLEC16A gene was evaluated with specific attention to the rs2903692 locus. The results showed that the frequency of the G allele increased significantly in T1DM patients (OR 1.28, 95% CI [1.05–1.57], *P* = 0.017), suggesting that the rs2903692 locus of the CLEC16A gene was significantly correlated with T1DM susceptibility in the Japanese population. Awata et al. [6] further examined that locus in juveniles (age of onset age < 20 years) and adults and found that the single-nucleotide polymorphisms at that locus were not significantly different between the two groups (data not shown). In addition to the findings in the Japanese population, correlations between the susceptibility of T1DM and different polymorphisms in CLEC16A gene were found in other ethnic groups. Martínez et al. [12] analyzed 309 cases of Spanish T1DM patients and 532 healthy controls and found that the G allele at the rs2903692 locus of the CLEC16A gene was related to susceptibility to T1DM (63% versus 58% in healthy controls, *P* = 0.047). Zoledziewska et al. [13] studied 1037 cases of Italian Sardinian T1DM patients and 1706 healthy control and showed that the A allele at the rs725613, locus of the CLEC16A gene is related to susceptibility to T1DM (47% versus 43% in healthy controls, OR = 1.15, *P* = 0.0051).

In our present study, based on previous locus studies and the literature, 17 loci in the CLEC16A gene were selected for analysis and the correlation between the CLEC16A gene and T1DM susceptibility in Chinese children was analyzed. (Technical support was provided by Bioyong Technologies Inc., Beijing.) The results showed that among the 17 polymorphism loci of the CLEC16A gene, the frequencies of only two loci, namely, rs12921922 and rs12931878, were increased significantly in patients with T1DM, as shown in Table 2. The frequency distribution of the alleles at the other loci showed no significant difference between patients and healthy control groups, as shown in Table 3. The frequency of T allele at rs12921922 was 90.8% in patients and 85% in healthy control, a statistically significant difference (*P* = 0.044; <0.05), suggesting a correlation between the T allele at rs12921922 and T1DM susceptibility. Similarly, the frequency of the A allele at rs12931878 was 90.1% in patients and 84.0% in healthy controls, also a statistically significant difference (*P* = 0.0435; <0.05), suggesting a correlation between the A allele at rs12931878 and T1DM susceptibility. The loci of the CLEC16A gene correlating with increased susceptibility to T1DM reported in other countries, including rs2903692 in Japanese, and Spanish populations, rs725613 in the Italian Sardinian population, (as shown in Table 4) and rs12708716 in Americans, British, Norwegian, Belgian, and Swedish populations, were not related to T1DM susceptibility in Chinese population. The sample being not large enough may be one of the causes, while other reasons such as differences in allele frequencies or linkage structure among Chinese, Japanese, and European populations may also account for the results.

In conclusion, current studies have elucidated that the CLEC16A gene is correlated with T1DM susceptibility in Caucasian, Japanese, and Chinese populations, indicating that, in addition to previously known genes, including HLA, insulin gene, PTPN22, and CTLA4, the CLEC16A gene is a newly discovered T1DM susceptibility gene. However, current results suggest that the susceptibility loci are different in different ethnic groups and geographical areas. Future studies on more ethnic groups with larger sample sizes are required to further confirm this conclusion.

## Figures and Tables

**Table 1:** Primer sequences for the targeted loci.

SNP number	PCR primer 1	PCR primer 2	Extension primer
RS8051196	ACGTTGGATGACCTGGACATGCCACAAAAC	ACGTTGGATGCAAGAATGAACAGAAGCCAC	CCGCCCCTGCTGCCC
RS2903692	ACGTTGGATGCAAAAGTGAGTTAGCCCCAG	ACGTTGGATGGCTAAGAACTGGGAAATGGG	CCCAGGACACCATGAA
RS7195452	ACGTTGGATGCACCATTGTATTCCAGCTTG	ACGTTGGATGTAGGCATGAGTCACTGTGTC	CCCCAGCTTGGACGACA
RS12708716	ACGTTGGATGCACACTTCATCCTCACTGAC	ACGTTGGATGGTCTTCAGCTAGTCCTCTGG	GTGAAGCGGCTATTACT
RS12922318	ACGTTGGATGATGGTTCCTGTAACTCAGAC	ACGTTGGATGGTACCAGGTATTTGCTAAGG	TGTGAGGACTAAGGGAA
RS7198004	ACGTTGGATGTCTGTGTAACACCAAGGAGC	ACGTTGGATGCTTGAGACTTGGCTCTCTTG	ACATGGAATGAATTCGCC
RS13337334	ACGTTGGATGCTTCAGGAGAAGATGAGAGG	ACGTTGGATGACAGACAGCATCCATCAGAG	TGTCGACTAGGCTGCATTC
RS7201845	ACGTTGGATGTCCACCTCTCTACTGTACTG	ACGTTGGATGCTTTGGGCCAAGTATCATGC	TGTACTGTAAAGCTCGAATA
RS7192695	ACGTTGGATGCCTGTTTTCCAGGTGGTTAC	ACGTTGGATGCTGTTCAAGCAAAGCCTCTG	GAGTGGTTACTTAGCCAGAT
RS3960630	ACGTTGGATGGCTGACGATGCTATATCGAG	ACGTTGGATGACTAAACTGGAATCACGTTG	CCCCCAGCAGCCTGGGACCCG
RS17673553	ACGTTGGATGGGTCAGACCACAATCAACAG	ACGTTGGATGTAGCCTCCTGTTTCCTTTTC	GGAAAGGTATGAAATGCAAAC
RS12931878	ACGTTGGATGCAGAGACCACAGGTATGAAG	ACGTTGGATGCAGCTTGCAAACTGGAAAGG	GTTTTCTACCATTTAGCAAAAG
RS12921922	ACGTTGGATGCACAAGACCAGAAATGATCTC	ACGTTGGATGGCAGTATTTCTAAACCTCTG	TAGATGTTATTATCCCCATGTTA
RS725613	ACGTTGGATGAGGATGAGACGCTGCCTATC	ACGTTGGATGGTCAACTGGGCTCTATCAAG	GGAGGCCTATCAGTGTTTAGCAC
RS16958028	ACGTTGGATGACGCCCACACACAACTAAAC	ACGTTGGATGTGAGGTAGAAGTCCTGCCTG	CCCCACAACTAAACAAAACTAGGG
RS17684919	AACGTTGGATGCAACAGCAGTGGCCATTATG	ACGTTGGATGTAGCTGGGCCAGCATTTTAC	CCCTCCATTATGACACTACGATAAC
RS12917716	ACGTTGGATGCCAGAGAGAAGAAAGAAAGG	ACGTTGGATGATGTGCCAGGCTGCTATGAG	AAAGAGAAGATGAAAAAGAAAAAAG

**Table 2:** Frequency distribution of the alleles at the two loci of the CLEC16A gene with statistical significance in Chinese children with TIDM and healthy control groups.

Gene	Alleles	Patient (131)	Control (121)	*χ* ^2^ Chi-square	*P*	OR (95% CI)
RS12921922	C : T	24 : 238	36 : 204	4.06	0.044	0.57 (0.33, 0.99)
RS12931878	A : C	236 : 26	200 : 38	4.08	0.0435	1.72 (1.01, 2.94)

**Table 3:** Frequency distribution of the alleles at the 15 loci of the CLEC16A gene without statistical significance in Chinese children with TIDM and healthy control groups.

Gene	Alleles	Patient	Control	*χ* ^2^	*P*	OR (95% CI)
RS12708716	A : G	209 : 53	183 : 59	1.25	0.2627	1.27 (0.83,1.93)
RS12917716	C : G	97 : 165	99 : 143	0.8	0.3713	0.85 (0.59,1.22)
RS12922318	A : T	51 : 211	41 : 201	0.53	0.4638	1.18 (0.75,1.87)
RS13337334	C : G	46 : 216	54 : 188	1.79	0.1809	0.74 (0.48,1.15)
RS16958028	A : T	234 : 28	218 : 24	0.08	0.7766	0.92 (0.52,1.63)
RS3960630	A : C	66 : 196	53 : 189	0.75	0.3849	1.2 (0.79,1.81)
RS7192695	C : T	231 : 31	222 : 20	1.76	0.1846	0.67 (0.37,1.21)
RS7195452	C : G	233 : 29	216 : 22	0.45	0.5	0.82 (0.46,1.47)
RS7198004	A : G	183 : 79	166 : 76	0.09	0.7609	1.06 (0.72,1.54)
RS7201845	A : G	244 : 18	216 : 26	2.37	0.1238	1.63 (0.87,3.06)
RS8051196	C : G	20 : 242	19 : 223	0.008	0.9272	0.97 (0.50,1.87)
RS17673553	A : G	245 : 17	215 : 27	3.44	0.0637	1.81 (0.96,3.41)
RS1784919	C : T	245 : 17	228 : 14	0.11	0.7426	0.88 (0.43,1.83)
RS2903692	A : G	57 : 205	59 : 183	0.49	0.4844	0.86 (0.57,1.31)
RS725613	A : C	245 : 17	228 : 14	1.27	0.2633	1.27 (0.83,1.94)

**Table 4:** Loci in the CLEC16A gene related to susceptibility of T1DM in different ethnic groups.

	Susceptibility loci	Susceptible allele	*P*	OR (95% CI)
Chinese	rs12921922	T	0.044	0.57 (0.33–0.99)
	rs12931878	A	0.0435	1.72 (1.01–2.94)
Japanese	rs2903692	G	0.017	1.28 (1.05–1.57)
Spanish	rs2903692	G	0.047	0.81 (0.66–1)
Italian Sardinian	rs725613	A	0.0051	
